# A response to iron involving carbon metabolism in the opportunistic fungal pathogen *Candida albicans*

**DOI:** 10.1128/msphere.00040-25

**Published:** 2025-04-04

**Authors:** Ritu Garg, Zhengkai Zhu, Francisco G. Hernandez, Yiran Wang, Marika S. David, Vincent M. Bruno, Valeria C. Culotta

**Affiliations:** 1 Department of Biochemistry and Molecular Biology, Johns Hopkins University Bloomberg School of Public Health 171261, Baltimore, Maryland, USA; 2 Department of Microbiology and Immunology, University of Maryland School of Medicine 12264, Baltimore, Maryland, USA; CNRS-Inserm-Université Côte d'Azur, Nice, France

**Keywords:** iron, fungal pathogen, metabolism, pyruvate dehydrogenase, lipoylation

## Abstract

Iron (Fe) is an essential micronutrient, and during infection, the host attempts to starve pathogens of this vital element through a process known as nutritional immunity. Successful pathogens have evolved means to evade this attack, an example being *Candida albicans,* the most prevalent human fungal pathogen. When Fe-starved, *C. albicans* induces multiple pathways for Fe uptake using the SEF1 trans-regulator, and we now describe a previously unrecognized effect of Fe on *C. albicans* metabolism that occurs independent of SEF1. Specifically, Fe limitation leads to inhibition of pyruvate dehydrogenase (PDH) connecting glycolysis to mitochondrial respiration. PDH inactivation involves loss of the LAT1 catalytic subunit harboring a lipoic acid co-factor. Protein lipoylation is a Fe-S dependent process, and lipoylated alpha-ketoglutarate dehydrogenase is also inhibited in Fe-starved *C. albicans*. SEF1 does not protect against PDH inactivation, and despite SEF1 induction of Fe import genes, cellular Fe levels drop dramatically during chronic Fe starvation. Such loss of LAT1 and lipoylation is also seen in Fe-starved bakers’ yeast *Saccharomyces cerevisiae*. In both yeast species, glucose is diverted toward the pentose phosphate pathway (PPP) and PPP production of NADPH is increased in response to low Fe and PDH loss. Additionally, glucose consumption is lowered in Fe-starved *C. albicans*, and non-PDH alternatives to producing Ac-CoA are induced, including pyruvate bypass and fatty acid oxidation pathways. *C. albicans* can adapt well to the effects of micronutrient loss on cell metabolism.

**IMPORTANCE:**

We describe a new response to Fe-starvation in a fungal pathogen involving carbon metabolism. Pyruvate dehydrogenase (PDH) that is central to glucose metabolism is inactivated at the post-translational level in Fe-starved cells. Nevertheless, the fungal pathogen can thrive by activating backup systems for metabolizing glucose. Methods that inhibit these compensatory pathways for carbon metabolism may prove beneficial in future anti-fungal strategies.

## INTRODUCTION

The transition metal Fe serves as co-factor for numerous metalloenzymes and is a vital micronutrient for nearly all forms of life. During infection, the host withholds Fe from invading pathogens in an attempt to thwart microbial growth through a process known as nutritional immunity (1). The pathogen can counteract this attack by upregulating efficient methods of Fe acquisition, economizing use of the metal, and by altering metabolism to endure micronutrient scarcity. *Candida albicans,* the most prevalent human opportunistic fungal pathogen, experiences large fluxes in Fe availability inside its animal host. In the gastrointestinal tract where *C. albicans* is a commensal, the fungus is exposed to abundant Fe. However, once infectious, the animal host withholds Fe from the organism (2, 3). In a mouse model of disseminated *candidiasis*, blood Fe levels drop dramatically (4, 5), and as the fungus invades the kidney, Fe-exclusion zones develop in the kidney medulla at sites of fungal lesions (6). This is indeed a case of chronic Fe-starvation, as Fe deprivation persists for days during *C. albicans* invasion of the kidney (6). Despite this severe Fe-limited environment, *C. albicans* thrives, and its animal host ultimately succumbs to fatal *candidiasis*.

Much of the *C. albicans* adaptive response to Fe-starvation occurs at the transcriptional level using a fungal trans-regulator known as SEF1 and induction of Fe transport genes (2, 3, 7). SEF1 is part of a regulatory triad that also includes the SFU1 transcriptional repressor of Fe uptake that is active under high Fe, and the HAP43 transcriptional repressor of Fe storage and Fe utilization genes that is active with Fe-starvation (2, 3, 7, 8). SEF1 and HAP43, but not SFU1, are critical for *C. albicans* virulence in the disseminated *candidiasis* model, demonstrating the fungal need to counteract the host-imposed Fe-starvation stress state (3, 8). During Fe-starvation, SEF1 induces three pathways for Fe uptake including a reductive pathway for ionic Fe, and pathways for acquiring Fe from siderophores and heme (9–14). SEF1 also induces fungal secretion of flavins, as well as an extracellular Cu-only superoxide dismutase (SOD4) (15–17). Less is known, however, about the cell metabolism responses to Fe-starvation in *C. albicans*.

In the bakers’ yeast *Saccharomyces cerevisiae*, Fe-starvation has a significant impact on glucose metabolism, including down-regulation of genes for mitochondrial respiration and changes in glycolytic metabolites (18–20). Respiration is a major consumer of Fe, involving numerous Fe-S or heme enzymes, and shifts in glucose metabolism away from respiration may help withstand effects of Fe-limitation. However, *S. cerevisiae* is a Crabtree-positive yeast where glucose represses respiration, compared to Crabtree-negative *C. albicans* that continues to respire with high glucose. It is not clear whether the glucose metabolism effects of Fe-starvation in *S. cerevisiae* would apply to other yeast species such as *C. albicans*.

Here, we use RNA-seq and biochemical approaches to identify the effects of chronic Fe-starvation on *C. albicans*. Our studies reveal numerous aspects of carbon metabolism impacted. Central to our findings was a post-translational effect of Fe-starvation on pyruvate dehydrogenase (PDH). This enzyme that converts pyruvate to Ac-CoA for the tricarboxylic acid (TCA) cycle is inactivated during Fe-starvation through loss of the LAT1 catalytic subunit and its co-factor, lipoic acid. Cells can overcome loss in PDH production of Ac-CoA by alternative pathways for carbon metabolism.

## MATERIALS AND METHODS

### Strains and growth conditions

*C. albicans* and *S. cerevisiae* strains are described in Table 1. The *sef1∆/∆* mutant in SC5314 was provided by Joachim Morschhaeuser (16), and the *sef1∆/∆* mutant in SN250 was obtained from the Fungal Genetics Stock Center. *S. cerevisiae* strain BY4741 and mutant derivatives were purchased from Horizon Discovery, U.K.

**TABLE 1:** Fungal strains

Species	Relevant genotype	Strain	Genotype	Source/reference
*C. albicans*	WT	SN250	*leu2Δ::C.m.LEU2/leu2Δ::C.d.HIS1, his1Δ/his1Δ, arg4Δ/arg4Δ, leu2D/leu2Δ ura3Δ/URA3, iro1Δ/IRO1*	(3)
*C. albicans*	*sef1∆/∆*	SN250 *sef1∆/∆*	*sef1Δ::C.m.LEU2/sef1Δ::C.d.HIS1, his1Δ/his1Δ, arg4Δ/arg4Δ, leu2Δ/leu2Δ, ura3Δ/URA3, iro1Δ/IRO1*	(3)
*C. albicans*	WT	SC5314	Clinical isolate	
*C. albicans*	*sef1∆/∆*	SC5314 *sef1∆/∆*	*sef1∆::FRT/sef1∆::FRT*	(16)
*S. cerevisiae*	WT	BY4741	*MATa, his3Δ1, leu2Δ0, met15Δ0, ura3Δ0*	
*S. cerevisiae*	*lat1∆*	BY4741 *lat1∆*	*MATa, his3Δ1, leu2Δ0, met15Δ0, ura3Δ0, lat1::kanMX4*	Horizontal discovery
*S. cerevisiae*	*gcv3∆*	BY4741 *gcv3∆*	*MATa, his3Δ1, leu2Δ0, met15Δ0, ura3Δ0, gcv3::kanMX4*	Horizontal discovery
*S. cerevisiae*	*lip2∆*	BY4741 *lip2∆*	*MATa, his3Δ1, leu2Δ0, met15Δ0, ura3Δ0, lip2::kanMX4*	Horizontal discovery

Fe-depleted yeast nitrogen base media was prepared as described (21) and used either as-is (Fe-starvation conditions) or supplemented with 1.0 or 5 µM FeCl_3_ to create Fe-replete conditions. *C. albicans* SN250 and its *sef1Δ/Δ* derivative cultures were supplemented with 80 mg/L arginine, and *S. cerevisiae* cultures were supplemented with synthetic complete supplement mixture (Sunrise Science Products, #1300-030). Unless noted otherwise, all experiments involved 10 mL cultures grown at 30°C to stationary phase at 200 rpm and chronically starved for Fe through either 6–7 average cell doublings (inoculated at OD_600_ of 0.05 and grown for 24 hours) or 12–13 average cell doublings (inoculated at OD_600_ of 0.001 and grown for 48 hours), with similar results. When Fe-replete, log phase *C. albicans* WT and *sef1∆/∆* cells exhibit mean doubling times of ≈80 minutes, while Fe-starved *sef1∆/∆* grows slower with a mean doubling time of ≈225 minutes compared to ≈115 minutes for WT. *S. cerevisiae* strains were also starved for Fe using YPD media (1% yeast extract, 2% peptone, and 2% dextrose) containing 100 µM of the Fe-selective chelator bathophenanthroline disulfonate (BPS, Sigma-Aldrich B1375-1G), and by inoculating at OD_600_ of 0.001 and 24 hour growth (nine average cell doublings).

### RNA analysis by RNA-seq and qRT-PCR

For RNA-seq, SN250 and the isogeneic *sef1∆/∆* mutant were grown as described above in Fe-deplete and Fe-replete cultures seeded at OD_600_ of 0.001. A total of 10–20 OD_600_ cell units were harvested, lysed by bead homogenization, and RNA isolated by RNAeasy Plus (Qiagen) (22). RNA samples were subjected to paired-end sequencing on an Illumina HiSeq 2500 (Novogene Inc.). Reads were aligned to the reference genome of *C. albicans* strain SC5314 by HISAT2 (23) and results used to create read counts for each open reading frame (ORF). Statistical analysis of differential gene expression involved the Bioconductor DE-seq package (24) and results of individual ORFs are in Table S1. Differentially expressed genes in Fe-starved versus Fe-replete conditions were analyzed using VolcaNoseR (25) with thresholds of log_2_ fold changes (LFC) of ≥1.0 and ≤−1.0 and adjusted *P*-values of ≤0.01. For volcano plots, differentially expressed genes with false discovery rates (FDR) of zero were given the same −log FDR values of 304.7 and 291.7 in the case of WT and *sef1∆/∆*, respectively (Tables S2 and S3). Gene set enrichment analysis was performed using shinyGo 0.81 (26) with LFC of >1.0 or <−1.0, and adjusted *P*-values <0.01.

For quantitative reverse transcription polymerase chain reaction (qRT-PCR), *C. albicans* or *S. cerevisiae* cells seeded at OD_600_ of 0.05 were grown in Fe-deplete and Fe-replete media as described above. Ten OD_600_ cell units were harvested, washed twice in diethylpyrocarbonate (DEPC)-treated water, and subjected to acid phenol extraction of mRNA (21). DNase treatment, cDNA synthesis, and qRT-PCR were carried out precisely as described (21). Amplicons of ≈200 bp were generated using primers described in Table 2. Values were normalized to *C. albicans TUB2* or *S. cerevisiae ALG9*.

**TABLE 2:** Gene primers used for qRT-PCR

Gene name	Primer direction	Sequence
Ca TUB2	Forward	GAG TTG GTG ATC AAT TCA GTG CTA T
	Reverse	ATG GCG GCA TCT TCT AAT GGG ATT T
Ca PDX1	Forward	AAG GCC ACC ATT GAT GTC GAA G
	Reverse	AAC TTC ACG TGG TGC TGA TTG T
Ca LAT1	Forward	ATG CTG GTG CTA AAG ACG TTC C
	Reverse	TGG AGC TGA AGT GGA GGT AGA A
Sc ZWF1	Forward	TAA GCC CGC CTA CGT GGA TG
	Reverse	CAT CAT GAT GGG GAC GCC CT
Sc ALG9	Forward	CCG TTG CCA TGT TGT TGT ATG
	Reverse	GCC AGG AAA TTG TAC GCT AAA

### Measurements of intracellular Fe

Intracellular Fe was measured in *C. albicans* inoculated at OD_600_ of 0.05 and grown in Fe-deplete and Fe-replete media as described above. A total of 25 OD_600_ cell units were harvested, washed in 10 mM Tris, 1 mM EDTA, pH 8.0, twice in milli-Q water, and digested in 600 µL 20% Optima grade nitric acid (Fisher Scientific A467-500). Samples were diluted to 6% nitric acid and Fe measured by quantitative inductively coupled plasma mass spectrometry using an Agilent 8900 triple quadrupole instrument (University of Maryland, School of Pharmacy, Mass Spectrometry Center). As an independent method, Fe from Fe-replete cells was measured by a BPS assay (27) with identical results, using cells digested in 300 µL 20% nitric acid and diluted to 3% nitric acid.

### Immunoblot studies

Immunoblots used cell lysates prepared precisely as described (21) from ≈10 OD_600_ cell units. An amount of 15–20 µg of lysate protein was subjected to denaturing gel electrophoresis on 4%–12% Bis-Tris polyacrylamide gels (Novex), and transferred to a PVDF membrane using the iBlot system. Primary antibodies included mouse anti-pyruvate dehydrogenase E2/E3bp (Abcam, ab110333) diluted 1:5,000, rabbit anti-lipoic acid (Abcam, AB58724) diluted 1:2,000, a rabbit PDH E1 alpha antibody (Proteintech, 18068-1-AP) diluted 1:2,000, or rabbit anti-SOD2 antibody at 1:1,000. Secondary antibodies were either anti-mouse antibody conjugated to IRDye 680RD (Licor 926-68070) or anti-rabbit antibody conjugated to IRDye 800CW (Licor 926-32211) at 1:10,000 dilutions. Blots were imaged by Odyssey (LI-COR Biosciences).

### Biochemical assays

To measure culture glucose, *C. albicans* was inoculated at an OD_600_ of 0.001 in Fe-replete or Fe-deplete media, and at designated time points, cells were removed by centrifugation and glucose measured in 5 µL spent media using the QuantiChrom glucose assay kit per manufacturer’s instructions (DIGL-100, Bioassay systems).

PDH and alpha-ketoglutarate dehydrogenase (KGD) activities were measured in crude mitochondrial lysates prepared from yeast cells seeded at either OD_600_ 0.001 or 0.05 and grown to OD_600_ 4–7 or 3–5 in the case of *C. albicans* or *S. cerevisiae,* respectively. A total of 100–200 OD_600_ cell units were harvested, washed with milli-Q water and 0.1 M Tris-SO_4_, 10 mM DTT, pH 9.4 (Tris-DTT buffer). Cells were resuspended at 0.2 g cells/mL in Tris-DTT buffer and incubated 30 minutes at 30°C. Cells were pelleted, washed in 1.2 M sorbitol, 20 mM KPO_4_, pH 7.4, and resuspended in the same buffer at 0.33 g cells/mL. An amount of 0.20–0.25 mg/mL lyticase was added (Sigma-Aldrich, L4025-100KU) and samples incubated at 37°C for 1 hour at 100 rpm. Spheroplasts were harvested by centrifugation at 1,000 × *g* for 5 minutes and resuspended in ice-cold 0.6 M sorbitol, 20 mM K^+^HEPES, pH 7.4, at a concentration of 0.5 g cells/mL based on original cell pellet weight. Samples were subjected to Dounce homogenization for 20–25 strokes, and crude mitochondrial pellets collected by 10 minutes centrifugation at 12,000 × *g*, resuspended in an equal volume of 50 mM KPO_4_, 0.25% Triton-X-100, pH 7.4, 1 mM PMSF, 1× protease inhibitor and lysed by bead homogenization. Lysates were clarified by centrifugation at 14,000 × *g*. PDH and KGD assays (28, 29) used 50 µg lysate protein in 200 µL of 50 mM KPO_4_, pH 7.4, 1 mM MgCl_2_, 2.6 mM cysteine, 2.5 mM NAD^+^ and containing either 2 mM thiamine pyrophosphate plus 2 mM pyruvate for PDH, or 0.2 mM thiamine pyrophosphate and 2 mMα-ketoglutarate for KGD. Coenzyme A (0.13 mM; C4780-25MG, Sigma) was added, and NADH formation over 10 minutes at 24°C was monitored at 340 nm in a UV-transparent flat bottom 96-well plate (Corning, #3635) using a Biotek Synergy HT plate reader.

Assays for pentose phosphate pathway production of NADPH were adapted from previous methods (30, 31). *C. albicans* or *S. cerevisiae* cells were inoculated at OD_600_ 0.05 or 0.001, respectively, and cultured as described above. Ten OD_600_ cell units were harvested, washed with milli-Q water, and resuspended in 150 µL 100 mM KPO_4_, pH 7.4, 2 mM MgCl_2_, 1× protease inhibitor, and 1  mM PMSF. Cells were lysed by bead homogenization. 2.0 µg lysate protein was added to 200 µL 50 mM Tris-HCl, pH 8.0, 5 mM MgCl_2_, and 0.4 mM NADP^+^. Upon addition of 5 mM glucose-6-phosphate (ZWF1 substrate), NADPH formation over 10 minutes at 30°C was monitored at 340 nm as described above. Since ZWF1 catalysis provides the 6-phosphogluconate substrate for GND1 (see Fig. 6A), the NADPH formed reflects activity from both enzymes (30, 31).

## RESULTS AND DISCUSSION

### Fe-starvation responses in WT and *sef1* mutants as seen in RNA-seq

**Fig 1:**
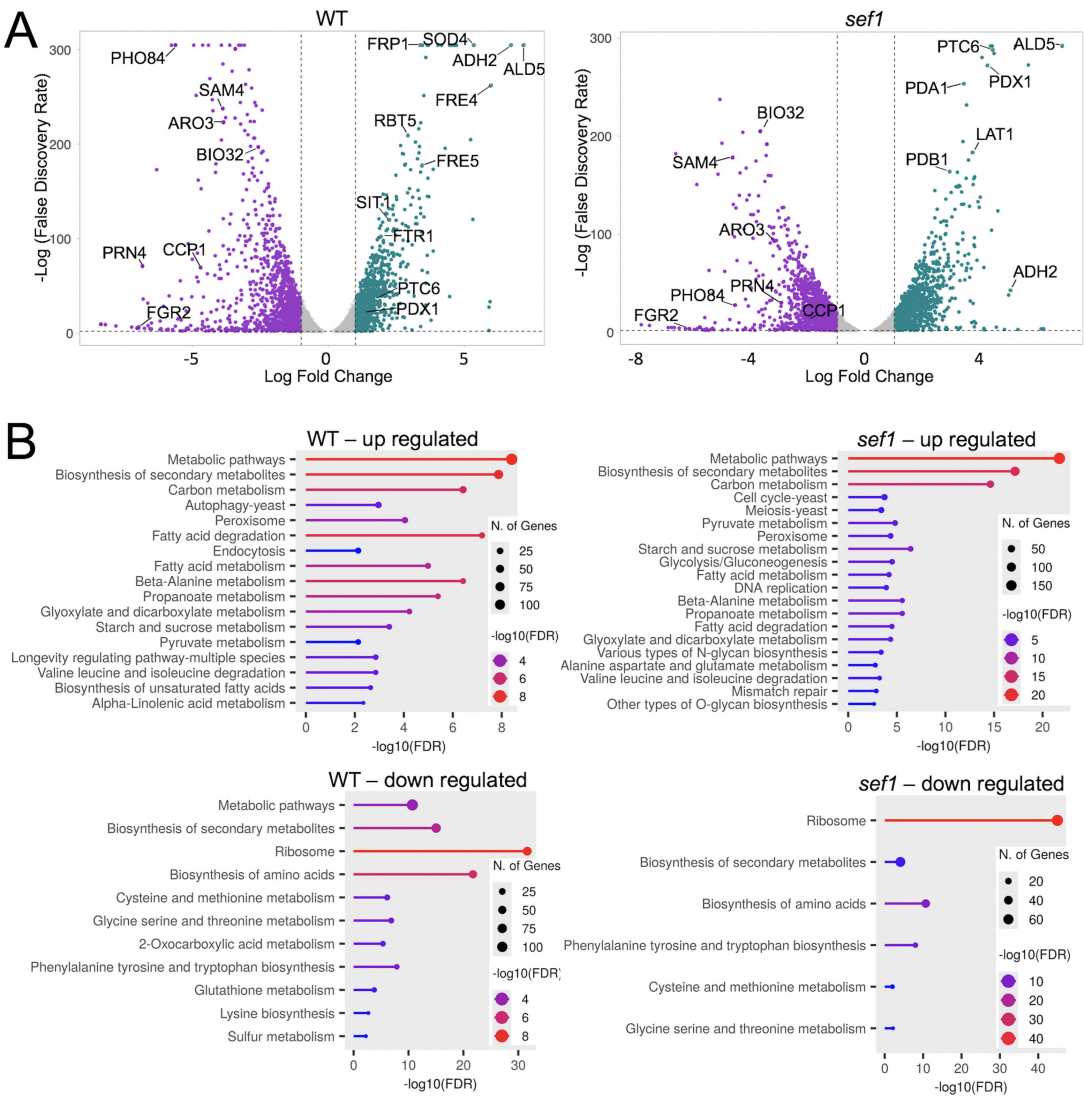
RNA-seq analysis of Fe-starved versus Fe-replete *C. albicans* wild-type and *sef1∆/∆* strains. The indicated cells were cultured in Fe-starvation and Fe-replete conditions prior to RNA-seq analysis as described in Materials and Methods. Shown are (A) volcano plots and (B) GO analyses of genes differentially expressed under Fe-starvation versus Fe-replete conditions for WT (left) and *sef1∆/∆* (right) strains. (A) Genes indicated are representatives of (i) those repressed by Fe-starvation in both WT and *sef1∆/∆* mutants including amino acid biosynthesis (*ARO3* and *SAM4*), Fe utilization (*BIO32, CCP1, PRN4*), and phosphate uptake (*PHO84* and *FGR2*) genes; (ii) genes induced by Fe-starvation in both WT and *sef1∆/∆* strains including *ALD5* and *ADH2* involved in carbon metabolism; (iii) genes induced by Fe-starvation in WT, but not *sef1∆/∆* mutants including those for Fe uptake (*FRP1, FRE4, FRE5, RBT5, SIT1, FTR1*) and *SOD4* superoxide dismutase; (iv) genes more strongly induced in Fe-starved *sef1∆/∆* cells including PDH subunits and its positive regulator (*PDX1, PDA1, LAT1, PDB1, PTC6*). (B) GO analysis was carried out by ShinyGo as described in Materials and Methods. Thresholds of LFC ≥1.0 and −1.0 and *P*-values of ≤0.01 were used to denote differentially expressed genes. Strains utilized: SN250 WT and the *sef1∆/∆* derivative.

An RNA-seq approach was used to identify the global mRNA response to Fe-starvation in *C. albicans* wild-type versus *sef1Δ/Δ* mutant cells. These studies used cells chronically exposed to Fe-starvation conditions by long-term growth (12–13 average cell doublings) in synthetic medium depleted of Fe. As controls, cells were cultured in the same media supplemented with low µM ferric Fe salts to create Fe-replete conditions. Results of genes differentially expressed in Fe-starvation versus Fe-replete conditions are summarized in the volcano plots and gene ontology (GO) analyses of Fig. 1, and findings with individual genes are in Table S1. We focused on gene expression changes using conventional thresholds of adjusted *P*-values of <0.01 and LFC of >1 or <−1. As seen in Fig. 1A, Fe-starved WT *C. albicans* cells exhibit the anticipated up-regulation of genes involved in uptake of Fe through reductive Fe import (*FTR1, FRE4, FRE5*), siderophores (*SIT1*) and heme (*FRP1, RBT5*), consistent with previous studies (3, 32). There was also strong induction of the Fe-regulated Cu-only superoxide dismutase *SOD4* (Fig. 1A) (17). As expected from previous studies (3, 17), none of these changes were observed with *sef1∆/∆* mutants (Fig. 1A). While this work was in progress, Xiong et al. analyzed the *C. albicans* transcriptome response to relatively short-term Fe-starvation (4 hour treatment with Fe chelators) and report an intriguing effect of Fe-limitation on DNA damage response genes (32). We observe similar effects in cells chronically starved for Fe, with numerous DNA damage repair genes expressed at higher levels in *sef1∆/∆* cells compared to WT (*RAD10, RAD51, RAD52, RAD7, CSM3, TOP3, MEC3, RFA2,* and *MPH1*0; Table S1).

Examination of genes repressed by Fe-starvation reveals evidence for protein translational suppression, as genes for ribosomal proteins and amino acid biosynthetic pathways were strongly down-regulated by Fe-starvation (Fig. 1A and B; Table S1). Studies in *S. cerevisiae* have also noted repression of amino acid synthesis with Fe-starvation (18, 19). Additional repressed loci in *C. albicans* encode processes that consume Fe such as genes for biotin synthesis (*BIO2, BIO32*), mitochondrial heme proteins *CCP1, YHB1,* and *CYC1* and all four Fe-binding pirins (*PRN1–PRN4*) (33, 34) (Fig. 1A; Table S1). Similar effects are evident in *sef1∆/∆* mutants (Fig. 1A; Table S1), and many of the down-regulated genes are known targets of HAP43 repression (35). Notably, there was pronounced repression of phosphate import genes *PHO84* and *FGR2* (36) in Fe-starved WT and *sef1∆/∆* cells (Fig. 1A). These transporters are not known to be targets of HAP43 repression. Intracellular phosphate can form insoluble complexes with ferric Fe in yeast mitochondria and vacuoles (37); thus, down-regulating phosphate import should enhance Fe availability in Fe-starved *C. albicans*. Repression of phosphate transporters was not evident in previous studies that used relatively short-term (4 hour) Fe-starvation (32), and may be an effect of long-term Fe-starvation stress.

Lastly, our RNA-seq analysis revealed certain impacts on glucose metabolism, including pyruvate metabolism as evident in GO analyses (Fig. 1B). Subunits of PDH and its positive regulator PTC6 were up-regulated by Fe-starvation in both WT and *sef1∆/∆* cells, and in fact, the effects were most pronounced in *sef1∆/∆* mutants (Fig. 1A). To our knowledge, such an effect of Fe on PDH has not been previously described and became the focus of our investigations.

### Subunits of PDH and its positive regulator are highly induced in Fe-starved *sef1∆/∆* mutants

**Fig 2:**
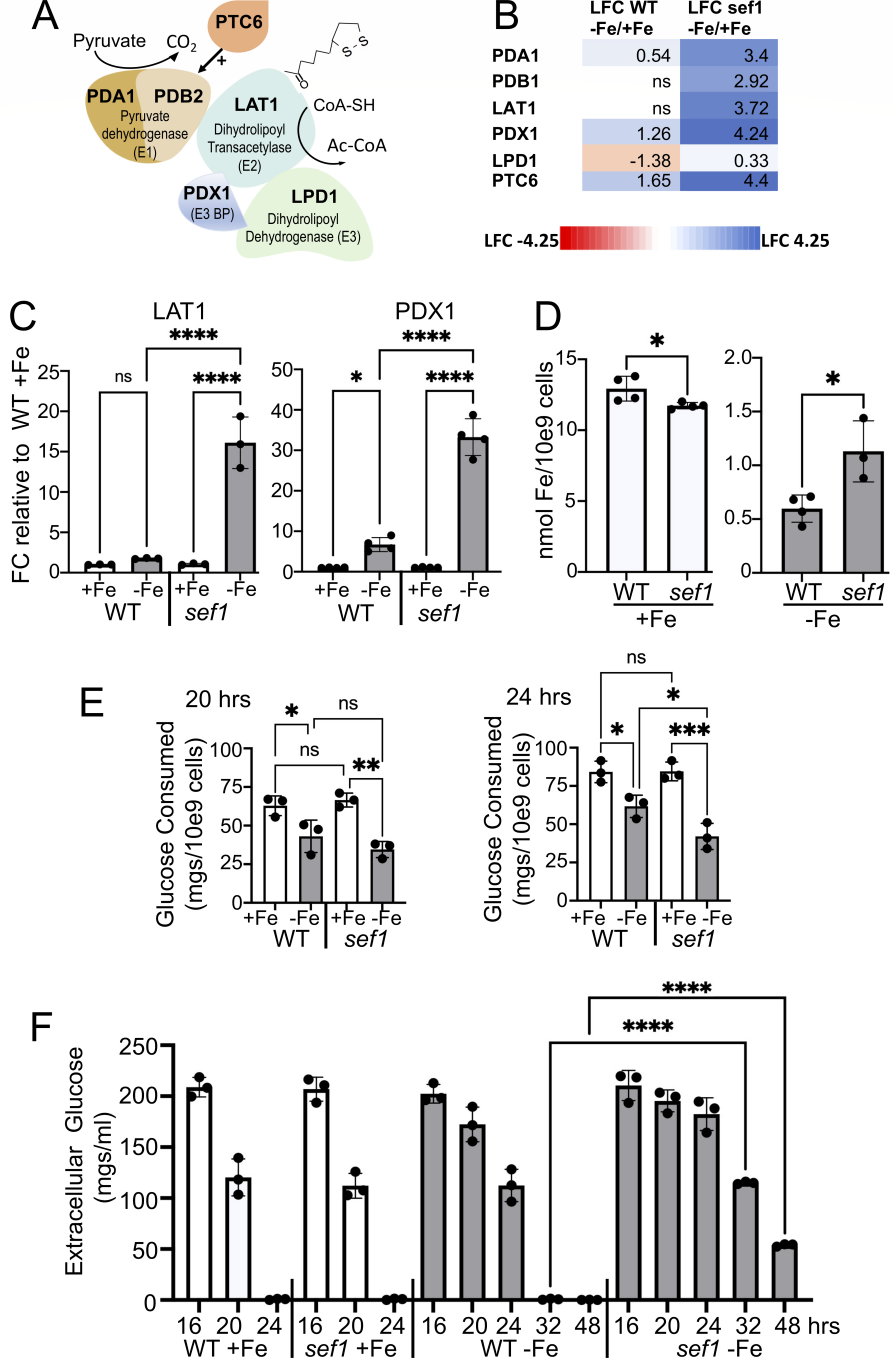
Induction of PDH genes in Fe-starved *C. albicans sef1∆/∆* mutants. (A) Cartoon depicting *C. albicans* PDH subunits catalyzing the conversion of pyruvate to Ac-CoA using a lipoic acid co-factor on LAT1. The phosphatase PTC6 positively regulates PDH by removing an inhibitory phosphate on E1. (B) Heat map analysis of PDH subunit genes and the PTC6 regulator. Values are LFC in gene expression from Fe-starved versus Fe-replete (−Fe/+Fe) WT and *sef1∆/∆* cells as determined from RNA-seq. (C and D) WT and *sef1∆/∆* cells cultured in Fe-replete (+Fe) or Fe-starvation (−Fe) conditions over 6–7 cell doublings were analyzed for (C) *LAT1* and *PDX1* expression by qRT-PCR, where values represent fold change over WT +Fe, or (D) intracellular Fe levels as described in Materials and Methods. (E and F) Glucose was measured in the extracellular media of the designated cultures as described in Materials and Methods. (E) When normalized to cell number, the glucose consumed by 20 and 24 hours is lower in Fe-starved versus Fe-replete cells. (F) The residual extracellular glucose when cultures reach stationary phase is higher in Fe-starved cells, particularly in *sef1∆/∆* mutants. Graphs in panels C–F show results from three to four independent fungal cultures; bars are mean and standard deviation. Statistical significance was tested by one-way analysis of variance (ANOVA) (C and E) or *t*-tests (D and F). ****P < 0.0001, ***P < 0.001, **P < 0.01, *P < 0.05, ns *P* > 0.05. Strains utilized: (B) *C. albicans* SN250 and the isogenic *sef1∆/∆* mutant; (C–F) *C. albicans* SC5314 and isogenic *sef1∆/∆* mutant.

PDH catalyzes the conversion of pyruvate to acetyl-coenzyme A and is comprised of three catalytic subunits: E1 (*C. albicans* PDA1 and PDB1), E2 (LAT1), and E3 (LPD1), and a non-catalytic E3 binding protein (PDX1) (Fig. 2A). The phosphatase PTC6 activates PDH (28, 38). With the exception of LPD1, all PDH subunits and the regulator PTC6 were highly induced in Fe-starved *sef1∆/∆* mutants, with smaller effects in WT cells (Fig. 2B). This up-regulation of PDH genes in the SN250 strain background (Fig. 2B) was reproduced in the independent SC5314 background (Fig. 2C) and in cells starved for Fe over either 12–13 (Fig. 2B) or 6–7 cell doublings (Fig. 2C).

We tested whether the high PDH expression of *sef1∆/∆* mutants correlated with a more severe Fe-starvation state. Intracellular Fe levels were found to be similar in WT and *sef1∆/∆* cells under Fe-replete conditions (Fig. 2D), and when Fe-starved, Fe levels dropped 10–20 fold (Fig. 2D). Surprisingly, *sef1∆/∆* mutants did not accumulate lower Fe, and if anything, Fe was higher in Fe-starved *sef1∆/∆* cells (Fig. 2D). The rationale for this elevated Fe is unclear but does not involve induced expression of known Fe import genes. In the defined media used here, cells acquire Fe through the reductive pathway, and all the pertinent genes are either repressed or unchanged in Fe-starved *sef1∆/∆* compared to WT cells (Table S1). Any effect on reductive Fe import would need to occur at the post-mRNA level. In any case, low intracellular Fe does not account for high PDH gene expression in Fe-starved *sef1∆/∆* mutants.

In *C. albicans,* PDH subunits are positively regulated by glucose and the GAL4 regulator (39–41). We therefore examined glucose levels. As seen in Fig. 2E, cellular glucose consumption is lowered in Fe-starved versus Fe-replete cultures, with a somewhat stronger effect in *sef1∆/∆* cells. Accordingly, extracellular glucose levels are higher in Fe-starved cultures, particularly in *sef1∆/∆* cells (Fig. 2F). These glucose effects may account for high PDA expression in Fe-starved *sef1∆/∆* cells. However, we cannot exclude the possibility that as a transcription factor, SEF1 directly or indirectly represses PDH gene expression.

### Effects of Fe-starvation on PDH protein

**Fig 3:**
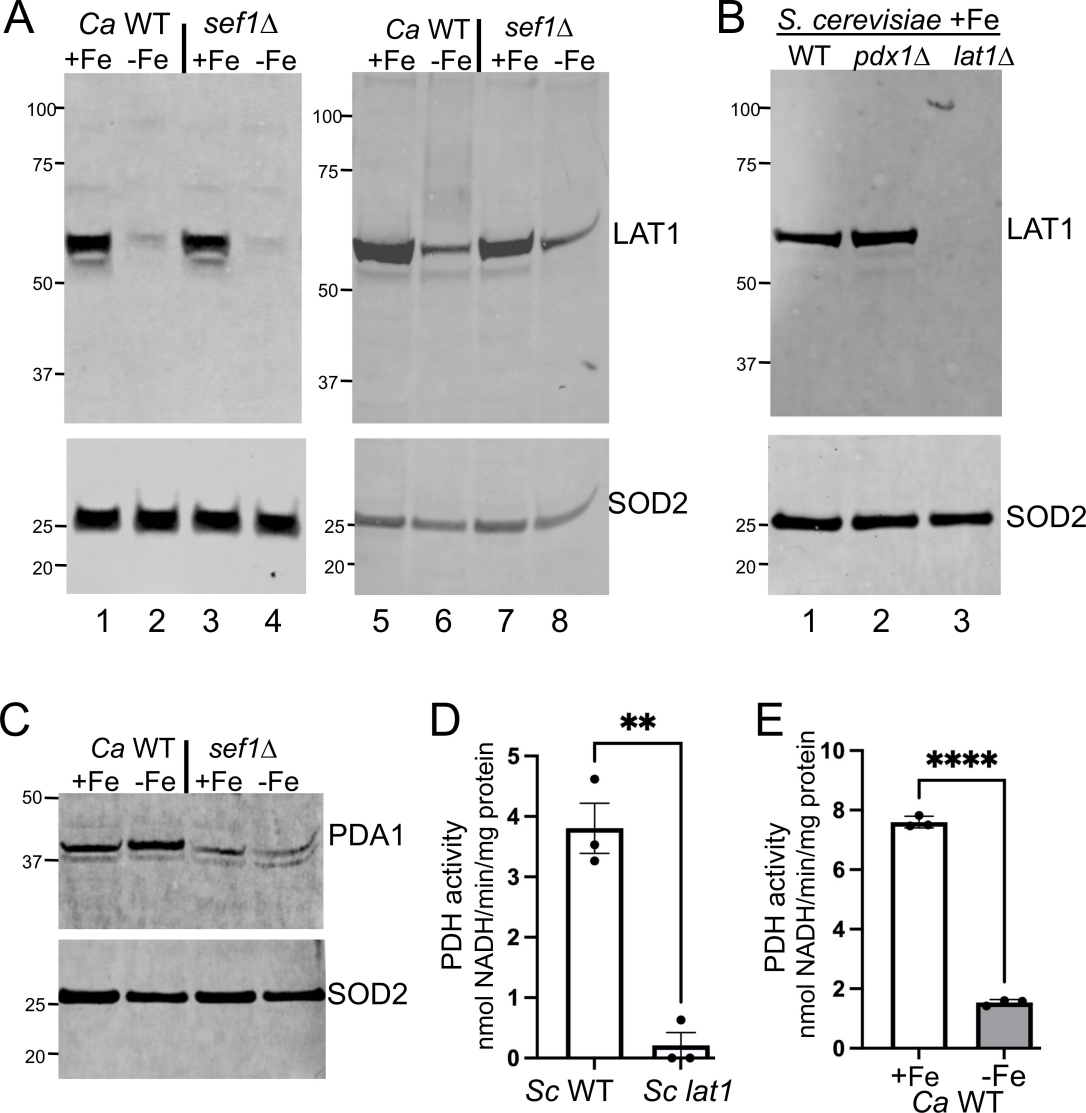
Down-regulation of *C. albicans* LAT1 with Fe starvation. (A–C) Immunoblots of fungal LAT1 (A and B) or PDA1 (C) were carried out in the indicated strains of either *C. albicans* (“*Ca”* A and C) or *S. cerevisiae* (B). Cells were cultured in Fe-replete (+Fe) or Fe-starvation (−Fe) conditions over 12–13 (A, lanes 1–4) or 6–7 doublings (A, lanes 5–8, B, and C) as described in Materials and Methods. Mitochondrial SOD2 is used as loading control, and molecular weight markers are indicated on the left. (D and E) PDH activity was measured in lysates of the indicated strains of *S. cerevisiae* (“*Sc*”) cultured under Fe-replete conditions (D), or of WT *C. albicans* cultured under Fe-replete (+Fe) or Fe-starvation (−Fe) conditions as described in Materials and Methods. Results are shown for three independent cultures; significance determined by *t*-tests. ****P < 0.0001, **P < 0.01. Strains utilized: *C. albicans* WT and *sef1∆/∆* mutant in the SC5314 background and *S. cerevisiae* BY4741 WT and mutant derivatives.

To investigate the effects of Fe-starvation on PDH protein, we used a mouse antibody widely used to study mammalian DLAT, the E2 PDH subunit with ~50% identity to *C. albicans* LAT1. This antibody detects a ≈60 kDa migrating protein in *C. albicans* (Fig. 3A). Although larger than the anticipated 50 kDa *C*. *albicans* LAT1, *S. cerevisiae* LAT1 is also known to migrate aberrantly at 60 kDa (42), and the anti-E2 antibody recognizes the same ≈60 kDa migrating species in *S. cerevisiae* (Fig. 3B). Importantly, this band was eliminated in a *S. cerevisiae lat1* mutant*,* but not *pdx1* mutant (Fig. 3B), confirming its identity as fungal LAT1.

Using the LAT1-reactive antibody, we examined the effects of Fe-starvation. Unexpectedly, and in contrast to mRNA results (Fig. 2B and C), *C. albicans* LAT1 protein levels were downregulated in Fe-starved WT and *sef1∆/∆* cells (Fig. 3A). The same results were observed in cells starved for Fe over either 12–13 cell doublings (Fig. 3A, lanes 1–4) or 6–7 cell doublings (lanes 5–8). To examine an additional PDH subunit, we employed an anti-E1 alpha antibody that detects a protein of the predicted size of 44 kDa *C*. *albicans* PDA1 (Fig. 3C). There was no downregulation of this protein in Fe-starved WT cells (Fig. 3C). Although overall protein levels were somewhat lower in *sef1∆/∆* cells for reasons that are unclear, the protein was not lost with Fe-starvation (Fig. 3C). How Fe-starvation impacts other PDH subunits is not known, yet even if these polypeptides are elevated in accordance with mRNA results (Fig. 2B and C), loss of LAT1 by itself should impede PDH activity. LAT1 is essential for PDH catalysis, and as demonstrated in an *in vitro* PDH assay, a *S. cerevisiae lat1* mutation severely inhibits PDH activity (Fig. 3D). Using this same *in vitro* assay in *C. albicans,* PDH activity is greatly decreased in Fe-starved cells (Fig. 3E), consistent with loss in LAT1 protein (Fig. 3A).

**Fig 4:**
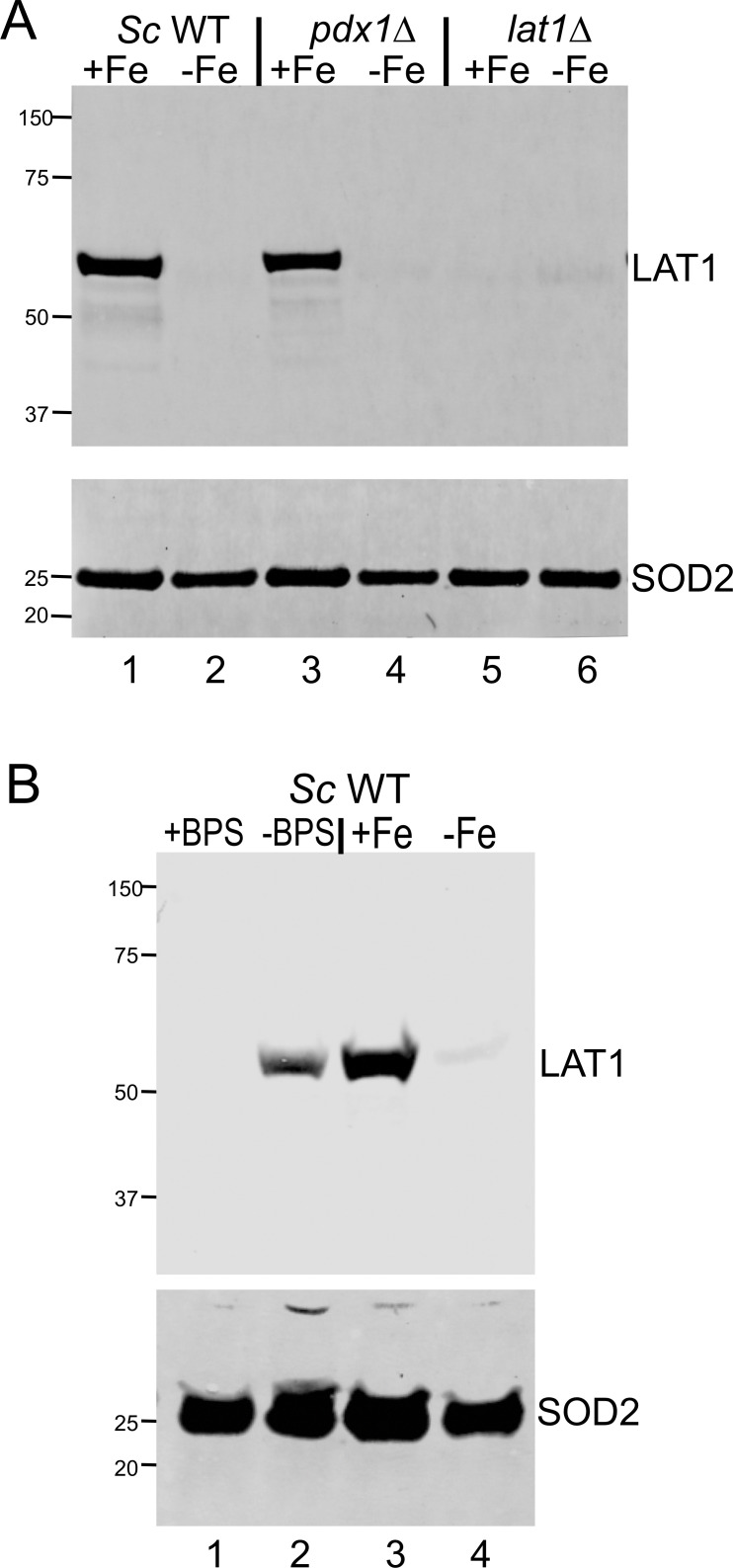
Down-regulation of *S. cerevisiae* LAT1 with Fe starvation. Immunoblot analysis of *S. cerevisiae* (“*Sc*”) LAT1 was carried out in the indicated strains grown either in Fe-replete (+Fe) or Fe-depleted (−Fe) minimal media (A and B, lanes 3 and 4), or in YPD enriched media supplemented where indicated with 100 µM of the Fe-selective chelator BPS (B, lanes 1 and 2). Mitochondrial SOD2 is used as loading control, and molecular weight markers are indicated on the left.

To our knowledge, this is the first report of Fe-starvation inhibiting PDH, and we tested whether this was specific to *C. albicans*. As seen in Fig. 4A *S*. *cerevisiae* starved for Fe also exhibits a loss in LAT1. The same results are obtained with WT cells and a *pdx1* subunit mutant, and in cells starved for Fe by growth in either Fe-deficient minimal media (Fig. 4A and B, lanes 3 and 4), or in enriched media treated with the BPS Fe-chelator (Fig. 4B, lanes 1 and 2). Interestingly, previous studies in *S. cerevisiae* have shown that Fe-starved cells accumulate high levels of pyruvate through an unknown mechanism (20). It is quite likely that this pyruvate hyperaccumulation resulted from PDH inhibition through loss of LAT1.

### Fe-starvation and protein lipoylation in yeast

**Fig 5:**
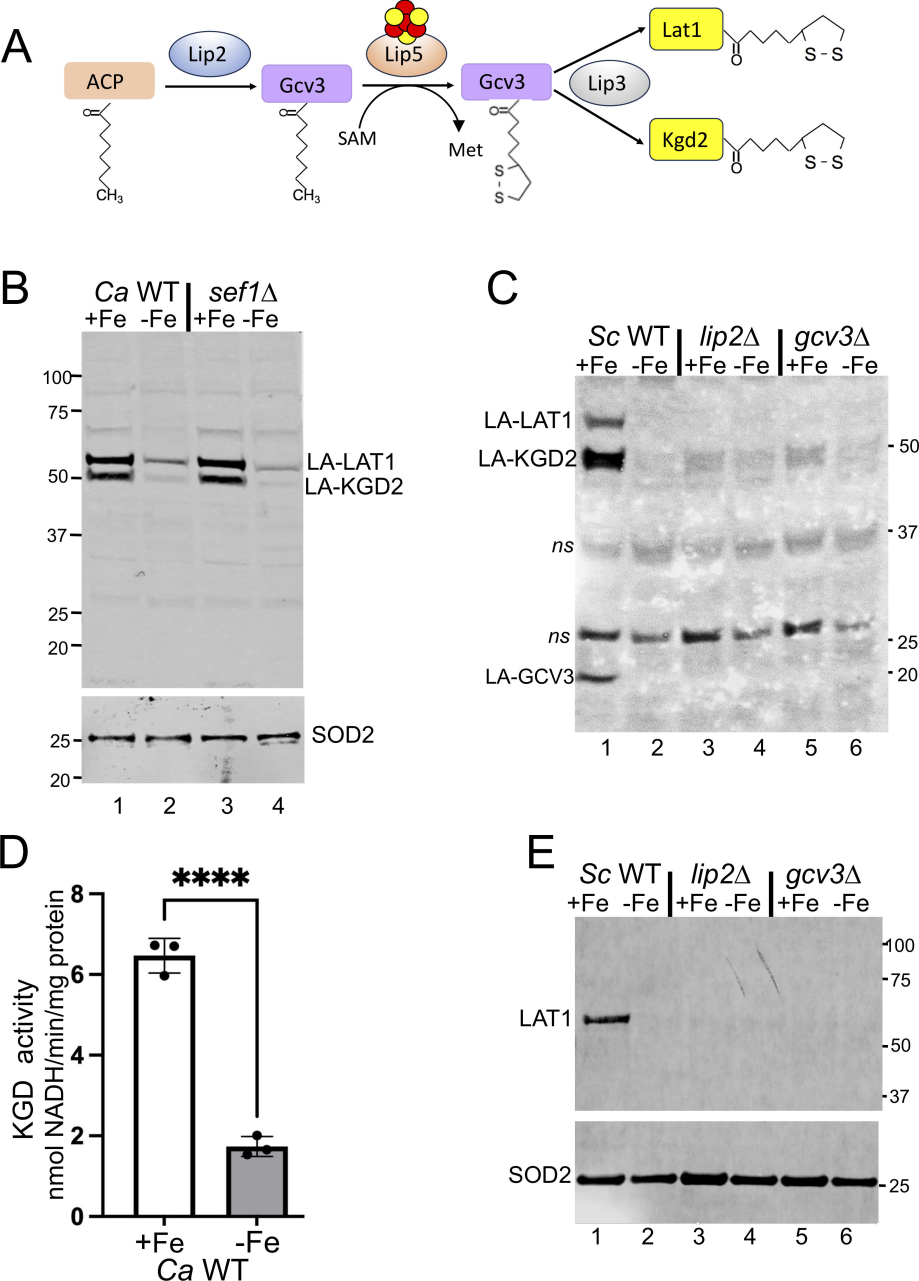
Effects of Fe limitation on protein lipoylation in *C. albicans* and *S. cerevisiae*. (A) Illustrated is the pathway for lipoylation of yeast GCV3, LAT1, and KGD2. The octanoyl transferase LIP2 transfers the octanoyl moiety from the acyl-carrier protein to GCV3, which is then converted to lipoic acid via the lipoyl synthase LIP5. Lipoic acid is then transferred to LAT1 and KGD2 by the lipoyl transferase LIP3 (42, 43). The sulfur for lipoic acid synthesis comes from the Fe-S cluster of LIP5 depicted in yellow and red balls (44). (B, C, and E) Cells were grown under Fe-replete (+Fe) or Fe-starvation (−Fe) conditions for 12–13 doublings as in Fig. 3A prior to immunoblot analysis of either lipoylated proteins in the indicated strains of *C. albicans* (B) and *S. cerevisiae* (C), or of LAT1 in *S. cerevisiae* strains (E). (B and C) An anti-lipoic acid antibody detects lipoylated (“LA-”) LAT1 and KGD2 in both yeasts, as well as lipoylated GCV3 in *S. cerevisiae. ns* indicates non-specific reactive bands in *S. cerevisiae* used as loading controls. SOD2 is also used as a loading control, and vertical numbers indicate molecular weight markers. (D) KGD enzymatic activity in lysates of Fe-replete (+Fe) or Fe-starved (−Fe) *C. albicans* was monitored as described in Materials and Methods. Shown are results from three independent cultures; significance determined by *t*-tests. ****P < 0.0001. Strains utilized: *C. albicans* WT and *sef1∆/∆* mutant in the SC5314 background and *S. cerevisiae* BY4741 WT and mutant derivatives.

The co-factor for LAT1 is lipoic acid, a sulfur-containing fatty acid. LAT1 is one of three lipoylated proteins in yeast, the others being the E2 subunit for KGD2 and the glycine cleavage H protein (GCV3). These proteins are lipoylated in a pathway involving yeast LIP2, LIP3, and LIP5 (Fig. 5A) (42, 43). Notably, the lipoyl synthase LIP5 contains an Fe-S co-factor (Fig. 5A), and its activity is also stimulated by a Fe-S ferredoxin (44, 45). Because of this dependence on Fe-S clusters for lipoic acid synthesis, we examined protein lipoylation under Fe-starvation conditions.

Lipoylated LAT1 and KGD2 can be visualized as ≈60 and ≈50 kDa migrating proteins on anti-lipoic acid immunoblots in *C. albicans* (Fig. 5B, lane 1) and *S. cerevisiae* (Fig. 5C, lane 1), consistent with previous studies (21, 42, 46). Lipoylated GCV3 is also prominent in *S. cerevisiae* (Fig. 5C, lane 1) (42, 43, 46), but not in *C. albicans* (Fig. 5B)*,* perhaps due to its low abundance. Importantly, lipoylation appears dramatically reduced in both yeast species under Fe-starvation conditions. *C. albicans* WT and *sef1∆/∆* cells exhibit similar losses in lipoylated LAT1 and KGD2 (Fig. 5B)*,* and in *S. cerevisiae,* Fe-starvation mimics effects of Fe-replete *lip2* and *gcv3* lipoylation mutants, and detectable lipoylation of all proteins is dramatically reduced (Fig. 5C). Lipoylation is necessary for yeast PDH and KGD activity (43), and accordingly, Fe-starvation leads to a great loss in *C. albicans* KGD activity (Fig. 5D), similar to the effects of Fe-starvation on PDH activity (Fig. 3E).

Total LAT1 protein is very low in Fe-starved cells (Fig. 3A and 4), which may explain low detection of lipoylated LAT1. However, based on the Fe requirement for lipoylation (44, 45), Fe-starvation is expected to impede LAT1 lipoylation, and the loss in LAT1 protein may be secondary to this post-translational defect. To test this hypothesis, we addressed whether lipoylation inhibition influences LAT1 protein under Fe-replete conditions using the *S. cerevisiae lip2* and *gcv3* lipoylation mutants. As seen in Fig. 5E, *lip2* and *gcv3* mutants have undetectable LAT1 protein even under Fe-replete conditions, mimicking the effects of Fe-starved WT cells. Inhibition of LAT1 lipoylation somehow triggers loss or turnover of the protein in yeast. This phenomenon may not be widespread, since mammalian DLAT protein levels do not decrease with lipoylation inhibition (45, 47).

Altogether, the studies of Fig. 2 to 5 reveal two independent effects of Fe-starvation on *C. albicans* PDH. First, there is induction at the mRNA level of PDH subunits and the PTC6 regulator that is particularly strong in Fe-starved *sef1∆/∆* mutants. Secondly, there is a dramatic loss in LAT1 protein. Although these findings appear contradictory, increases in mRNA do not always result in increased protein at steady state (48). Any effects of elevated mRNA on LAT1 protein synthesis may be nullified by LAT1 turnover triggered by lipoylation inhibition or other Fe-starvation effects.

### Implications for loss of PDH and Fe-starvation in carbon metabolism

**Fig 6:**
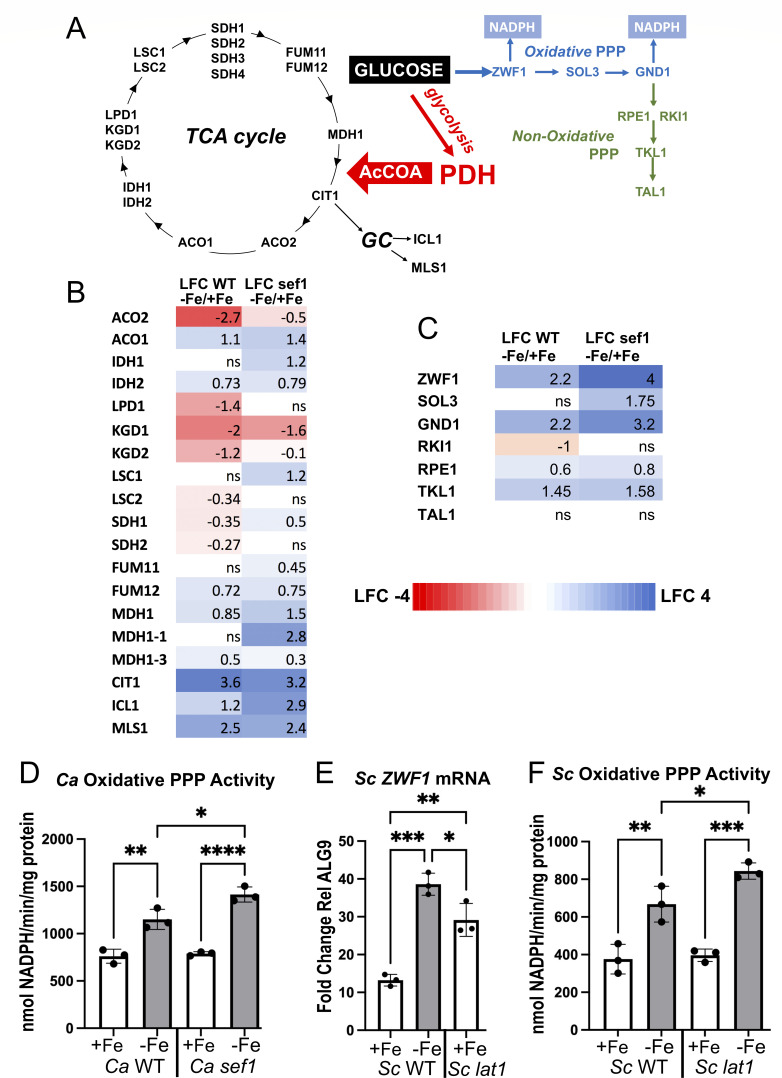
TCA cycle, glyoxylate cycle, and pentose phosphate pathway effects of Fe-starvation. (A) Shown are pathways for glucose metabolism either through glycolysis and the TCA and glyoxylate cycles ("GC"), or through the pentose phosphate pathway (PPP) including both oxidative and non-oxidative PPP. See text for details. (B and C) Heat map analyses of TCA and glyoxylate cycle genes (B) and of PPP genes (C). Values are LFC in gene expression from Fe-starved versus Fe-replete (−Fe/+Fe) WT and *sef1∆/∆* cells as determined from RNA-seq. (D and F) A cell-free assay for oxidative PPP that monitors glucose 6-phosphate stimulated production of NADPH was carried out with the indicated strains of *C. albicans* (D) or *S. cerevisiae* (F) grown under Fe-replete (+Fe) or Fe-starved (−Fe) conditions as described in Materials and Methods. (E) *ZWF1* mRNA was measured by qRT-PCR in the indicated strains of *S. cerevisiae* grown under Fe-replete (+Fe) or Fe-starved (−Fe) conditions as in Fig. 3B and compared to *ALG9* as housekeeping gene. Graphs of D–F show results from three independent cultures and statistics analyzed by one-way ANOVA. Comparison of *Sc* WT and *Sc lat1* −Fe in part F was also analyzed by *t*-test (*P* = 0.047). ****P < 0.0001, ***P < 0.001, **P < 0.01, *P ≤ 0.05.

PDH is central to glucose metabolism in that it connects glycolysis to the TCA cycle and mitochondrial respiration. We therefore examined several aspects of carbon metabolism in Fe-starved *C. albicans*. The TCA cycle consumes Fe based on Fe-S cluster requirements of aconitase and succinate dehydrogenase, and in *Aspergillus fumigatus* and *S. cerevisiae,* TCA cycle genes are repressed during Fe-starvation (20, 49). Through RNA-seq analysis, Fe-starvation coincided with a marked repression of *C. albicans* KGD subunits and of Fe-S ACO2, but otherwise there was no global repression of the TCA cycle (Fig. 6B). Moreover, similar trends were observed in WT and *sef1∆/∆* mutants, demonstrating that unlike *Lachancea kluyveri* yeast (50), *C. albicans* SEF1 is not a major regulator of TCA cycle genes. Interestingly, we observed a strong induction of *CIT1* in Fe-starved WT and *sef1∆/∆* mutants (Fig. 6B). The CIT1 citrate synthase also functions in the glyoxylate cycle that bypasses the CO_2_^−^ generating steps of the TCA cycle to produce C_4_ compounds for macromolecule synthesis (51). In *C. albicans,* the glyoxylate cycle also involves peroxisomal ICL1 and MLS1 (51) (Fig. 6A), both of which are induced in Fe-starved WT and *sef1∆/∆* cells (Fig. 6B). The glyoxylate cycle is up-regulated during Fe-starvation.

In addition to producing pyruvate through glycolysis, glucose can be converted to ribose sugars through the pentose phosphate pathway (PPP) (Fig. 6A). PPP has both an oxidative component where ZWF1 and GND1 generate NADPH for oxidative stress protection (52, 53), and a non-oxidative component that produces precursors for nucleotides, fatty acids, and amino acids. PPP is induced in yeast when glycolysis is disrupted (54, 55), and we tested whether PPP is also induced when PDH is inhibited by Fe-starvation. As seen in Fig. 6C, the NADPH-producing *ZWF1* and *GND1* genes of oxidative PPP are markedly induced in Fe-starved *C. albicans* WT and *sef1∆* cells. Using a cell-free assay to examine ZWF1 and GND1 production of NADPH, we observed elevated activity in Fe-starved compared to Fe-replete cells with somewhat higher levels of NADPH production in Fe-starved *sef1* mutants (Fig. 6D) consistent with transcriptome data (Fig. 6C). To test if loss of PDH triggers this induction, we analyzed effects of *lat1∆ S. cerevisiae* mutants. As with *C. albicans, ZWF1* mRNA and *in vitro* NADPH production are increased in Fe-starved WT *S. cerevisiae* cells (Fig. 6E and F). Moreover, *lat1* mutants show elevated *ZWF1* expression under Fe-replete conditions, akin to Fe-starvation effects (Fig. 6E). However, *lat1∆* mutations only marginally increased NADPH production in the cell-free assay (Fig. 6F), indicating that PDH loss may not be the only factor inducing PPP during Fe starvation.

**Fig 7:**
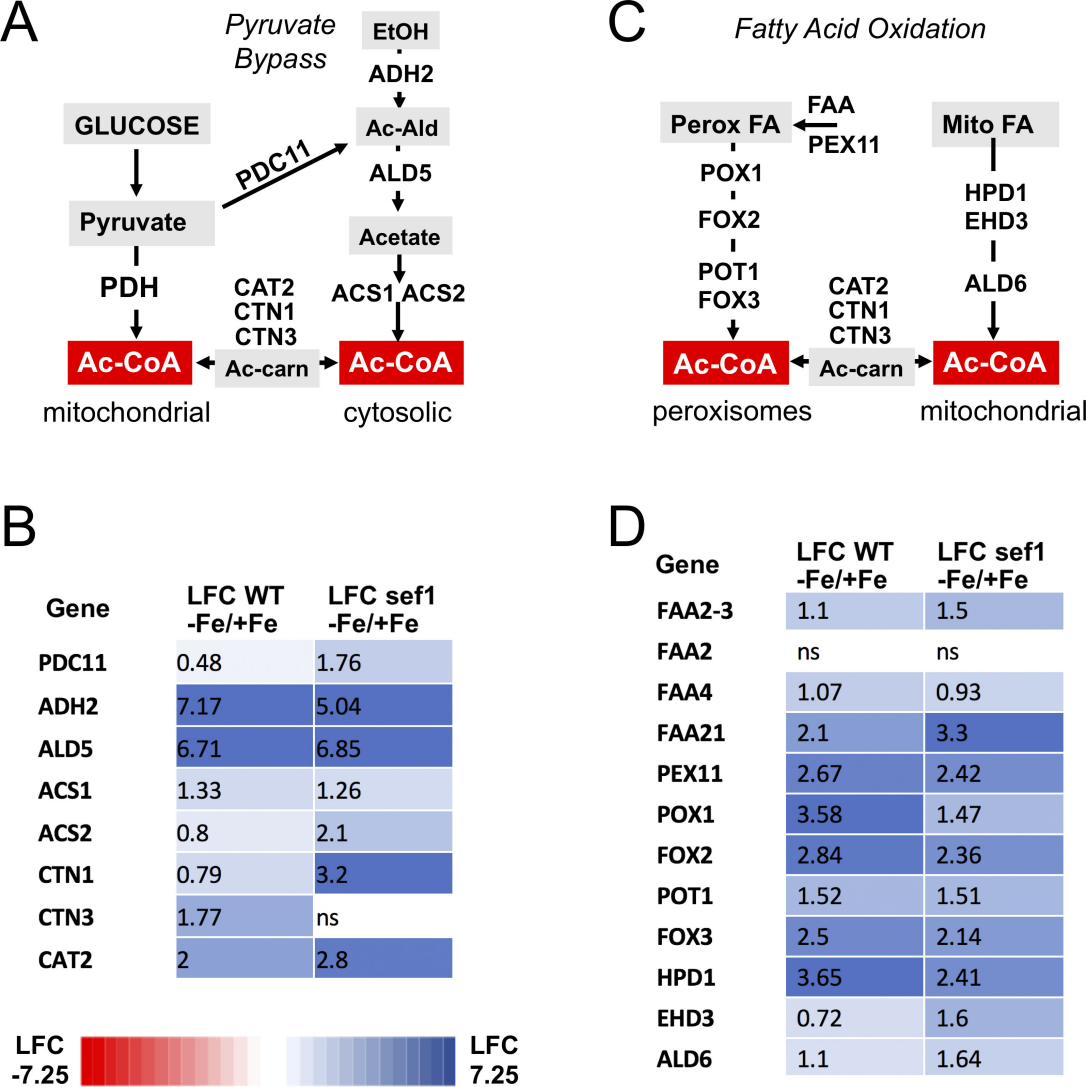
Pyruvate bypass and beta oxidation pathways for Ac-CoA. (A and C) Shown are pathways for non-PDH sources of Ac-CoA in (A) the cytosol through pyruvate bypass or (C) peroxisomes and mitochondria through fatty acid beta oxidation. (A) Pyruvate can lead to Ac-CoA production through either mitochondrial PDH or the cytosolic pyruvate bypass system involving pyruvate decarboxylase (*C. albicans* PDC11). Acetaldehyde (“Ac-Ald”) for cytosolic Ac-CoA can also originate from alcohol dehydrogenase (*C. albicans* ADH2). The cytosolic Ac-CoA can then be transported into the mitochondria by acetyl-carnitine (“Ac-carn”) transferases. (C) Beta oxidation of fatty acids begins with their transport into peroxisomes through FAA and PEX11 transporters, then metabolized ultimately to Ac-CoA through fatty acyl-CoA oxidases POX1, FOX2, POT1, and FOX3. *C. albicans* also has a mitochondrial system for fatty acid oxidation. See the main text for details. (B and D) Heat map analyses of genes for (B) cytosolic Ac-CoA production and Ac-CoA transport and (D) beta oxidation pathways for Ac-CoA production. Values are LFC in gene expression from Fe-starved versus Fe-replete (−Fe/+Fe) WT and *sef1∆/∆* cells as determined from RNA-seq.

The inhibition of PDH during Fe-starvation is expected to impact Ac-CoA, which is not only needed for the TCA cycle, but also for macromolecule synthesis and protein acetylation (56). However, Ac-CoA can also be produced in the cytosol through the pyruvate-bypass system (Fig. 7A). Here, pyruvate decarboxylase (*C. albicans* PDC11) converts pyruvate to acetaldehyde, then metabolized to acetate and Ac-CoA by aldehyde dehydrogenases and acetyl CoA synthases ALD5, ACS1, and ACS2 (Fig. 7A) (57–59). Yeast ADH2 also produces acetaldehyde for Ac-CoA (60), and the cytosolic Ac-CoA is then delivered to mitochondria through carnitine transferases CTN1, CAT2, CTN3 (Fig. 7A) (61, 62). All these genes for pyruvate bypass and Ac-CoA transport were up-regulated by Fe-starvation in *C. albicans* (Fig. 7B). In fact, *ADH2* and *ALD5* were the most robustly induced genes in Fe-starved WT cells, with LFCs of ≈7 (Fig. 1A and 7B; Table S1), and similar effects were seen with *sef1∆/∆* mutants (Fig. 7B). Ac-CoA can also be generated by peroxisomal fatty acid oxidation, and *C. albicans* has an additional branch of beta oxidation in the mitochondria involving fatty acyl oxidases HPD1 and EHD3 and a mitochondrial aldehyde dehydrogenase ALD6 (58) (Fig. 7C). Peroxisomal-mitochondrial exchange of Ac-CoA is mediated by the carnitine transferases (Fig. 7C). All these segments of beta oxidation production and transport of Ac-CoA are induced during Fe-starvation in both WT and *sef1* mutants (Fig. 7D). Together, the strong induction of pyruvate bypass and beta oxidation systems for Ac-CoA provide plausible mechanisms for how Fe-starved cells cope with PDH deficiency.

### Conclusions

Our studies have revealed a new response to Fe-starvation involving carbon metabolism. It has long been known that glucose metabolism in the mitochondria is impacted by Fe-limitation through loss of Fe-S and heme requiring enzymes for oxidative phosphorylation, and we now demonstrate that a central glucose metabolizing enzyme that has no Fe co-factor is also dramatically affected. Activity of mitochondrial PDH is greatly diminished during Fe-starvation due to loss of the LAT1 catalytic subunit, an effect of lipoylation inhibition and/or other consequences of Fe-limitation. Such inhibition of PDH during Fe-starvation is common to both the opportunistic fungal pathogen and Crabtree-negative *C. albicans* and the non-pathogenic Crabtree-positive *S. cerevisiae,* and may be widespread across fungal species exposed to chronic Fe-starvation as would occur inside an animal host. Even so, loss of PDH as a key source of Ac-CoA can be mitigated by enhancing various bypass pathways for producing Ac-CoA. Also induced by Fe-starvation are alternative methods for utilizing glucose through the PPP and for metabolizing Ac-CoA through the glyoxylate cycle. Such adaptations can help the organism survive during chronic Fe-starvation, as is seen when *C. albicans* invades the kidney during disseminated candidiasis (6). In this model, a number of changes in carbon metabolism are evident, including induction of genes for the glyoxylate cycle (*ICL1, MLS1*) and for non-PDH methods of producing Ac-CoA (*ADH2, CTN1, FOX2, HPD1*) (63), akin to responses observed here in Fe-starved cultures *in vitro*. Studies directed at inhibiting one or more of these compensatory carbon metabolism mechanisms for enduring Fe-starvation may prove beneficial in future therapeutic avenues for infectious fungi.

## SUPPLEMENTAL MATERIAL

Captions msphere.00040-25-s0001.pdf Captions for Table S1 to S3.

Table S1 msphere.00040-25-s0002.xlsx RNA-seq data, all comparisons.

Table S2 msphere.00040-25-s0003.xlsx Adjusted FDR for WT.

Table S3 msphere.00040-25-s0004.xlsx Adjusted FDR for *sef1*.

ASM does not own the copyrights to Supplemental Material that may be linked to, or accessed through, an article. The authors have granted ASM a non-exclusive, world-wide license to publish the Supplemental Material files. Please contact the corresponding author directly for reuse.
