# Supplementary material for: A response to iron involving carbon metabolism in the opportunistic fungal pathogen Candida albicans
Source: mSphere. 2025 Apr 4;10(4):e00040-25. doi: 10.1128/msphere.00040-25 (PMC12039268; doi:10.1128/msphere.00040-25)
Supplement: Captions — for Table S1 to S3. [file msphere.00040-25-s0001.pdf]

The Supplemental Material contains RNA-seq datasets

Table S1: RNA-seq data all comparisons
The LFC and FDRs for genes differentially expressed in Fe-starved versus Fe-replete WT strains and the *sef1* mutant.

Table S2: Adjusted FDR for WT.
Genes differentially expressed in WT cells with FDR values of zero were all assigned -log FDR values of 304.7 to include such genes in volcano plot analyses.

Table S3: Adjusted FDR for sef1.
Genes differentially expressed in *sef1* cells with FDR values of zero were all assigned -log FDR values of 291.7 to include such genes in volcano plot analyses.
